# FlowerPatch: New Method to Measure Nectar Volume in Artificial Flowers

**DOI:** 10.3390/insects16070714

**Published:** 2025-07-11

**Authors:** Edwin Lara-Perez, Jose Agosto Rivera, Tugrul Giray, Remi Megret Laboye, Edwin Flórez Gómez

**Affiliations:** 1 Department of Mathematical Sciences, University of Puerto Rico, Mayagüez Campus, Mayagüez 00680, Puerto Rico; edwin.lara@upr.edu; 2 Department of Biology, University of Puerto Rico, Rio Piedras Campus, San Juan 00931, Puerto Rico; jose.agosto1@upr.edu (J.A.R.); tugrul.giray@upr.edu (T.G.); 3 Depatment of Computer Sciences, University of Puerto Rico, Rio Piedras Campus, San Juan 00931, Puerto Rico; remi.megret@upr.edu

**Keywords:** nectar sensor, artificial flowers, nectar consumption, oscillator circuit, frequency signal, microcontroller, dynamic monitoring

## Abstract

This article proposes a new Flower Patch Nectar Sensor to address the problem of detecting and measuring nectar in artificial flowers used in experiments on pollinator behavior. Traditional methods have focused mainly on recording the visits of pollinators to the flowers, without addressing the dynamic variations in nectar in terms of volume and concentration. The proposed approach provides more detailed information about the nectar consumption by bees and allows for the determination of the optimal time to refill the flowers. This study introduces an innovative method that uses electrodes and an oscillator circuit to measure the volume of nectar present in the flower. The system correlates the concentration of nectar with a frequency signal that can be processed by a microcontroller. It was evaluated using initial volumes ranging from 1 μL to 4 μL, demonstrating its ability to accurately detect variations in nectar, even up to the point where the frequency approaches zero. The results confirm that this method allows us to identify how the reward offered to pollinators (represented by nectar) varies over time, in terms of concentration, under both controlled and natural conditions. Additionally, graphs are presented that show the relationship between an initial volume of 4 μL and variations in the frequency signal over a period of 25 min, highlighting the influence of these factors on nectar dynamics. This work not only introduces an innovative approach for the dynamic monitoring of nectar in artificial flowers but also lays the groundwork for future studies on the physical and chemical modeling of nectar in response to environmental conditions.

## Simple Summary

This study presents a new sensor for artificial flowers, designed to investigate nectar dynamics in relation to pollinator behavior. Other existing systems enable nectar delivery or the detection of pollinator visits. With our sensor, called FPNS, we contribute the ability to directly indicate the presence and variation of nectar. This allows us to determine if nectar may have been consumed or if its state was affected by environmental conditions. With the FPNS, we aim to optimize the manual processes of nectar replenishment and improve data collection in pollinator experiments.

## 1. Introduction

Interactions between bees and flowering plants involve complex processes of nectar collection, pollination, and foraging decision-making. Bees visit flowers primarily to obtain nectar, a reward whose availability significantly influences their behavior, visitation patterns, and pollination efficiency. These interactions are dynamic, with nectar volume and concentration fluctuating due to factors like bee consumption, evaporation, and environmental conditions. To effectively study these processes under controlled experimental conditions, researchers frequently employ artificial flowers, as discussed by Giray et al. [1].

Behavioral assays gather data by monitoring the visits of pollinators to the artificial flowers in terms of number, timing, and duration while controlling the quantity and concentration of the nectar available in a small well at the center of each flower. Honey bees visit the well and collect the nectar using their proboscis. Following Cakmak et al. [2], the typical protocol is to refill the well with nectar solution after each visit to ensure the consistent availability of nectar during an experiment.

Such behavioral assays are quite demanding in terms of visual observation and timely refill due to the fast moving insects, which has motivated the design of automated nectar delivery systems and automatic visit detection.

For example, research by Debeuckelaere et al. [3] and Kuusela and Lämsä [4] proposed the automation of nectar delivery and the detection of visits. The systems of Essenberg [5] and Sokolowski et al. [6] designed artificial flowers that deliver nectar in a discrete or controlled manner. Others, such as Moffatt et al. [7] and Keasar et al. [8], manipulate variables such as the reward rate, spatial arrangement of flowers, or metabolic conditions. However, this previous research has focused solely on measuring the number of visits to flowers, and the control of the nectar delivery, but has not addressed the variables that affect the amount of nectar available in them and its automatic measurement. Furthermore, recent computer vision approaches, such as that of Rodriguez-Cordero et al. [9], allow for real-time bee tracking and visit detection at the flower-patch but with the difficulty of independently detecting the presence of nectar or quantifying or estimating the volume consumed. Integrating a nectar detection sensor into this field offers the possibility of direct nectar detection, thus complementing visual tracking in flower patch experiments.

In this context, the present work stands out by proposing an innovative methodology that not only measures visits but also the volume of nectar remaining in artificial flowers. This allows for more precise data on bee behavior and nectar dynamics, which represents a significant advance in the understanding of nectar collection processes.

The second objective is to develop a model that can be used with sensor readings to improve the estimation of the presence or absence of nectar in artificial flowers.

## 2. General Architecture

**Figure 1:**
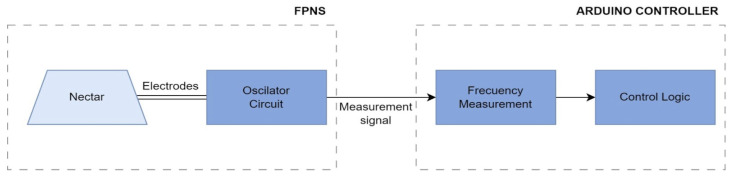
Block diagram of the nectar detection system correlating the volume of nectar with the output frequency of the oscillator circuit.

The nectar sensing system is composed of two key mechanisms: a Flower Patch Nectar Sensor (FPNS) that measures the amount of nectar in the nectary with a 555 oscillator circuit that converts the variation of the intrinsic resistance of the nectar into a frequency signal, and a microcontroller (such as the Arduino UNO) that interprets the signal and controls the detection process. This system focuses solely on the precise measurement of nectar in loop and its variation over time (see Figure 1).

The system is made up of the following essential components:

Flower Patch Nectar Sensor (FPNS): This sensor is composed of an artificial flower and a nectar detection circuit.

Flower and electrodes: The artificial flower provides the nectary with electrodes at the bottom coming into contact with the nectar. The electrodes are connected to the oscillator circuit to detects the intrinsic resistance of the nectar.
555 Oscillator and Detection Circuit: The 555 oscillator is configured in astable mode and acts as a pulse generator. The frequency generated is proportional to the resistance of the nectar present in the sensor. This circuit is designed with external resistors and capacitors to adjust the duty cycle and output frequency.

Microcontroller and Algorithm: An Arduino Uno was used to measure the frequency generated by the 555 oscillator. This microcontroller offers a simple and efficient way to capture these signals and send them for analysis, working at a clock speed of 16 MHz (16 million cycles per second), which is more than enough for basic tasks.

## 3. Materials and Methods

In this section, we present and discuss the details of each module and how they are validated experimentally.

### 3.1. Flower Design

In this study, artificial flowers are designed as flat plates with a central hole leading to the nectary, where trained bees access nectar. The FPNS sensor detects nectar absence when it is withdrawn. This type of bee interaction with the artificial flower is comparable to that in other autonomous floral patches, such as Debeuckelaere et al. [3].

**Figure 2:**
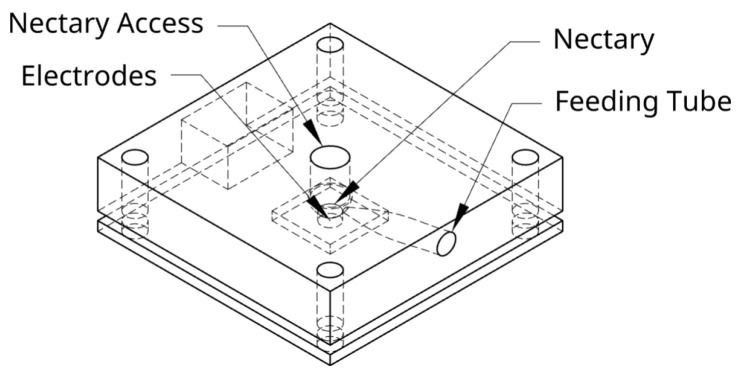
Artificial flower: design with PCB and lead-free electrodes, lateral wiring maintaining a flat nectary.

The artificial flower design consists of two parts as shown in Figure 2: a 3D-printed block for a removable top with the nectary hole in the middle, and a PCB with the electrodes at the bottom.

The removable top was modeled in OnShape (model available at [10]). It consists of a central nectary hole and a side tube through which the nectar is supplied. The design was exported as an STL file for 3D printing and replication. It was printed with a resin printer (Elegoo Saturn 8k, Shenzhen, China), thanks to its ability to manufacture parts with a precision of 0.0285 mm. The accessible side of the flower has an internal thickness of 1 mm, and this type of printer allows these high-precision tasks.

At the base of the flower are the electrodes that cross the nectary and maintain direct contact with the nectar for its measurement (see Figure 2). The model maintains this shape to prevent external factors such as wind from directly affecting the nectar and also to mitigate its impact somewhat due to natural conditions.

### 3.2. Circuit Board Design

The electrodes of the flower PCB are connected to an external curcuit board that drives the electrical signal of the electrodes and measures the reaction using an oscillatory circuit.

**Figure 3:**
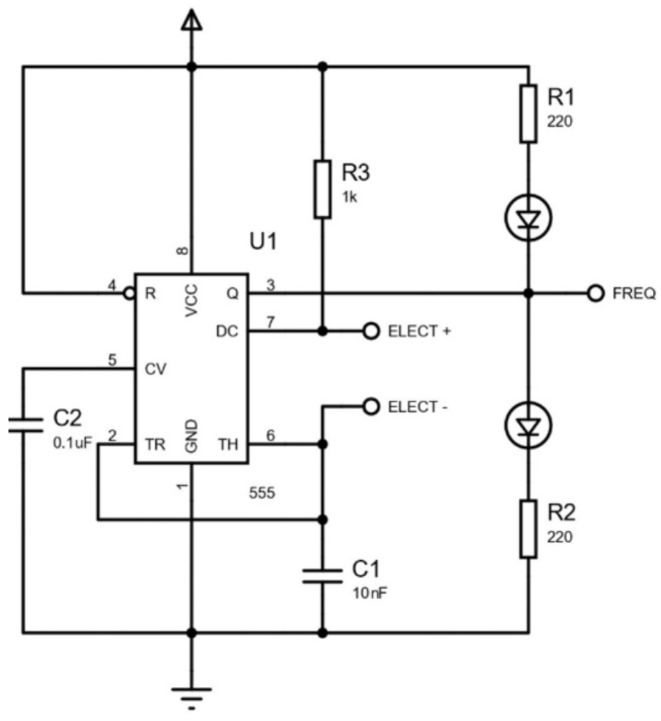
Oscillator circuit diagram for nectar detection: the circuit primarily uses a 1kΩ resistor, and two capacitors (one of 0.1 μF and another of 10 nF) to ensure an output frequency of approximately 200 Hz for a 4 μL nectar drop with an initial concentration of 2 M (molar), prepared by dissolving 69.45 g of sugar in 100 mL of water.

Figure 3 shows the diagram of the circuit. A 555 timer IC configured in astable mode was integrated into this sensor, using a 555 package, an external 1 kΩ resistor, 0.1 μF and 10 nF capacitors, and protoboard jumper wires, all controlled by an Arduino Uno, which was chosen for its low cost, ease of programming, and versatility due to its extensive library support. Power and ground were provided by connecting pin 8 (VCC) and pin 4 (RESET) of the 555 to the Arduino’s 5 V terminal, while pin 1 (GND) and one lead of each capacitor ($C_{1}$ and $C_{2}$) were tied to the Arduino’s ground. To set the oscillation frequency, resistor $R_{1}$ (1 kΩ) links VCC (pin 8) to pin 7 of the 555, and resistor $R_{2}$ (replaced by the electrodes) connects pin 7 to the common node of pins 6 (threshold) and 2 (trigger). Additionally, $C_{1}$ (0.1 μF) is placed between pin 6 and ground, and $C_{2}$ (10 nF) between pin 5 and ground. The output signal at pin 3 is routed to digital pin 2 of the Arduino to generate external interrupts, as specified in the implemented pseudocode shared at [11].

**Figure 4:**
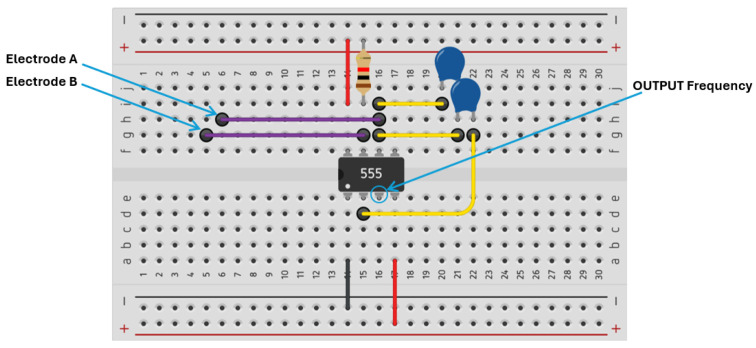
Design and visual representation of FPNS connections.

To develop the system, the FPNS detection circuit was first designed as a breadboard (see Figure 4) connected to the flower electrodes on one side and outputting a frequency for the Arduino on the other side.

### 3.3. Microcontroller Design

Frequency measurement is carried out through pin 2 of the Arduino, which is configured as an external interrupt. This allows for the detection of signal state changes (rising edges) from the 555 oscillator without requiring constant polling in the main program loop, thereby enabling accurate frequency measurement.

**Figure 5:**
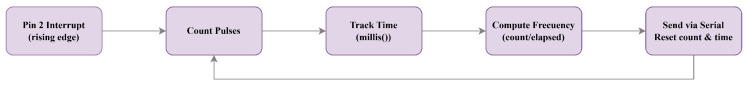
Block diagram of the loop program.

Each time a pulse is detected, a counter increments. After a predefined time interval (measured using an internal system timer that counts the milliseconds since the device was turned on), the frequency is calculated by dividing the number of pulses detected by the elapsed time (see Figure 5). The resulting data can be transmitted via serial communication to an external laptop for storage and further processing.

### 3.4. Experimental Design and Validation

Experimental validation of the FPNS system was conducted under controlled laboratory conditions (31 °C and 61% RH), which served as the basis for all measurements, without a loss of generality. The validation aimed to evaluate the sensor’s ability to detect changes in nectar concentration and volume in artificial flowers.

Two main experiments were performed for this validation. The first experiment evaluated the sensor’s temporal response and the effect of evaporation on frequency measurements. This test involved monitoring the sensor reading every second over a 25-min period for a 4 μL nectar droplet.

The nectar solution was prepared by dissolving 69.45 g of sugar in 100 mL of water (2 M), under ambient conditions maintained at 31 °C and 61% relative humidity.

The second experiment analyzed the FPNS’s sensitivity to gradual volume increases. This was conducted by adding successive 1 µL increments of nectar every 30 s until a total volume of 4 μL was reached, with the frequency being measured after each addition.

Each of these experiments was repeated three times under identical laboratory conditions to ensure data consistency. The same procedure was also replicated for different initial volumes, such as 6 μL, to confirm consistent behavior under similar conditions. All raw data and experimental logs can be found in the online repository [11] for further details.

## 4. Results

**Figure 6:**
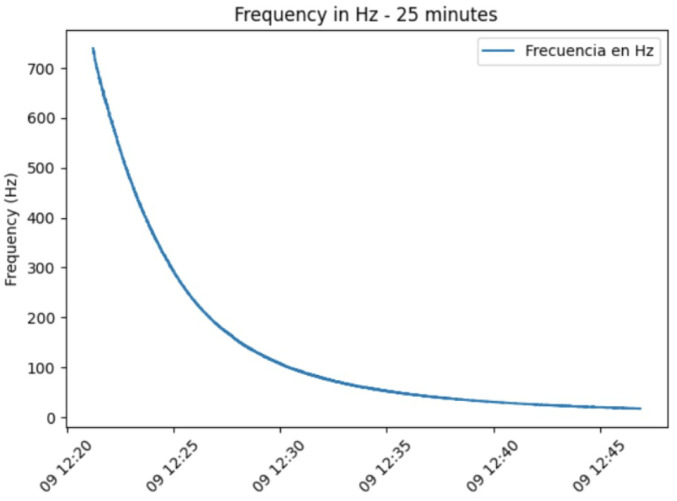
Nectar frequency over time for a 4 mL droplet.

Figure 6 illustrates the temporal behavior of the FPNS over a 25-min period following the application of a 4 mL nectar droplet. Frequency changes were recorded every second. The graph consistently shows a gradual decrease in frequency over time, starting from an initial frequency of approximately 850 Hz and steadily declining to around 0 Hz at the end of the observation period. This observed decline represents a total signal frequency reduction over 25 min.

**Figure 7:**
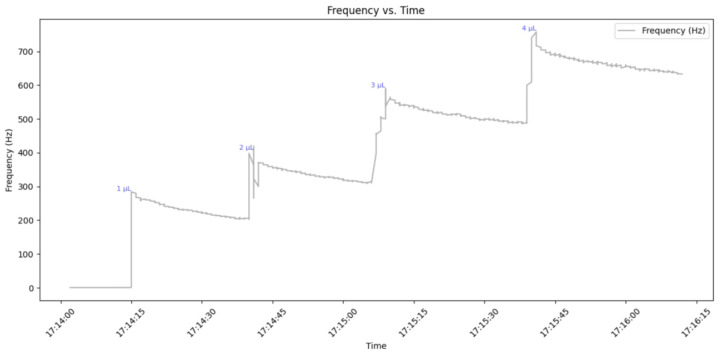
Frequency changes with incremental additions of 1 to 4 mL of nectar.

Figure 7 demonstrates the sensor’s response to the incremental addition of nectar volume. In this experiment, frequency output was recorded after successive 1 mL increments of nectar were added every 30 s, from an initial 1 mL up to a total volume of 4 mL. The frequency output consistently exhibits a distinct stepped pattern. For each 1 mL addition, the frequency increased, with distinct steps observed at 300 Hz for 1 μL, 410 Hz for 2 μL, 600 Hz for 3 μL, and 800 Hz for 4 μL. The sensor consistently recorded higher frequencies for larger volumes, demonstrating a clear and reproducible relationship between the applied nectar volume and the measured frequency.

## 5. Discussion

The observed gradual behavior demonstrates the sensor’s ability to accurately detect minute variations in nectar volume. This level of sensitivity is excellent for experiments seeking to replicate natural conditions, where dynamic fluctuations in nectar availability are common. Furthermore, the system’s demonstrated accuracy contributes to the development of more comprehensive models of nectar dynamics, significantly improving our understanding of the complex interactions between pollinators and plants.

The main challenge of this study was to build a suitable sensor capable of measuring or detecting the presence of nectar in the artificial flower, especially for small droplets of approximately 4 μL. Initial attempts to use standard components, such as the FC28 soil moisture sensor, proved unfeasible due to their excessive size and integration complexity. Therefore, a custom circuit was developed to handle low-level signals and accurately measure minute amounts of nectar. Leveraging fundamental principles of electronics and considering nectar as a variable “resistor” due to its low electrical conductivity, a sensor based on a 555 oscillator circuit was designed. This design converts variations in the intrinsic resistance of nectar into an oscillating output signal, which increases or decreases with the presence or absence of nectar. This approach culminated in the development of the FPNS sensor, a tool that leverages recent advances in sensor design and offers great potential for future autonomous systems, robotics, and equipment that require the precise measurement and detection of rewards such as nectar (including varying sugar concentrations). This methodology also presents a new avenue for addiction experiments in pollinator studies, for example, by monitoring substances such as alcohol in honeybees as by Giannoni et al. [12].

The results presented for the FPNS sensor demonstrate its capability to detect dynamic changes in nectar over time, rendering it suitable for continuous data collection and accurate presence/absence detection. The observed decrease in frequency, particularly relevant in the 25-min evaporation experiment, provides valuable real-time information for behavioral studies with bees, allowing for the estimation of nectar volume and concentration. Furthermore, by integrating computational methods, the FPNS system offers the potential for dynamically adjusting nectar availability, thereby optimizing the reward system for pollinators. This dynamic adjustment capability is especially relevant in natural environments, where variables such as temperature fluctuations and direct sun exposure directly impact nectar solutions.

Therefore, the proposed FPNS system provides information not previously available for automated nectar delivery, by detecting the presence of the nectar itself. Existing systems for nectar delivery either focus on the delivery itself, as proposed by Essenberg [5], Sokolowski and Abramson [6], Keasar [7], or use bee arrival and/or departure detection, as proposed by Debeuckelaere et al. [3], Moffatt [8] and Kuusela and Lämsä [4], but may not have enough direct evidence to determine whether a bee has partially or fully consumed nectar before refilling the well. The FPNS sensor provides crucial support by allowing for the integration of nectar status into predictive models for artificial flowers equipped with dedicated detection hardware. This feature is particularly beneficial as it now allows for integrating nectar presence/absence with other sensors and systems aimed at detecting bee visits and foraging behavior, helping to determine the nectar’s state and protecting it from abrupt changes caused by environmental exposure. This also provides a complementary signal to computer vision systems such as those proposed by Rodriguez et al. [13] and Rodriguez-Cordero et al. [9].

The FPNS sensor is designed for straightforward replication, as most of its key components lack defined polarity, simplifying assembly with basic electronics knowledge or by following the provided methodological instructions. To enhance practicality and facilitate the generation of multiple FlowerPatch units (e.g., as described by Lara-Pérez [11]), the proposed design also allows for the rapid cleaning of the nectar without compromising the electronics that is separated from the flower. The electrodes are integrated in the PCB and are easily cleaned and dried for storage without risk of damage. The modular design in two parts also facilitates the long-term maintenance of the flowers through the easy replacement of damaged parts.

Given that the nectar signal gradually decreases in frequency from the moment of application—a phenomenon influenced by environmental conditions causing evaporation or solidification—it becomes essential to integrate a mathematical model within the sensor system if the aim is to predict or accurately estimate the solution’s state over time. This temporal weakening of the signal is a direct consequence of the nectar’s susceptibility to variable environmental factors, specifically the temperature and relative humidity conditions replicated in these experiments. Therefore, future work will focus on integrating additional temperature and humidity sensors to model and compensate for such influences in real time, thereby extending the sensor’s reading range and enabling robust operation beyond controlled laboratory settings.

While the sensor can be adapted to different nectar volumes or rewards and was tested from 1 μL to 4 μL, it also has potential for larger quantities. Higher initial volumes are anticipated to yield a stronger, more stable signal with elevated frequency values. However, considering that the reward delivered to pollinators in these types of experiments is often limited, our focus remained on optimizing performance within the low-volume range. It should be noted that the current design presents some limitations, such as the necessity to readjust components for detecting even smaller nectar volumes. As per the manufacturer’s datasheet by Diodes Incorporated [14], the sensor output frequency is inversely proportional to both nectar resistance and capacitance; therefore, only by changing capacitors can the output frequency be affected. While the sensor’s intrinsic sensitivity cannot be varied by this means, the reading range can be extended by adjusting the frequency as previously mentioned. This ensures that variable environmental conditions outside the laboratory would begin to affect the nectar at higher initial frequency values, providing a broader spectrum for detecting changes before the signal reaches zero. Additionally, current system encodes nectar concentration into an oscillating signal whose frequency is decoded by the Arduino Uno on the binary valued digital input pin. Other design may be explored in the future to convert the oscillatory signal into a DC voltage signal electronically to simplify the software reading by using an analog input pin on the microcontroler side.

Future work will integrate temperature and humidity sensors to account for environmental influences or help estimate the current nectar state, aiming to improve measurement accuracy. The data obtained will support the development of mathematical models for nectar evaporation, freezing, and consumption, which can be combined to build autonomous systems. The strong correlation between frequency and volume identified in this work serves as a basis for developing advanced predictive models. These models can incorporate dynamic changes in nectar properties, such as evaporation, freezing, and consumption, which are challenging for autonomous systems to determine without human intervention. They can contribute to variables that are difficult to ascertain (e.g., nectar presence, absence, and amount consumed by bees), thereby offering valuable feedback for studies aiming to understand the reaction of pollinators to nectar status more accurately.

Furthermore, the FPNS will be applied in open-field conditions with freely flying, marked bees, connecting the sensors to a continuous data acquisition system during real foraging trials. By implementing automated systems with artificial intelligence that reads sensor signals and predicts overall system behavior, we can significantly enrich the understanding of nectar dynamics in pollinator behavior studies. These results thus open up new opportunities for more comprehensive and robust experiments, including integration with systems such as the system proposed by Rodriguez-Cordero et al. [9], allowing for dynamically linking variables such as the current nectar state with the identification and tracking of individual bee behavior using neural networks, as shown by Santiago et al. [15].

## 6. Conclusions

The Flower Patch Nectar Sensor (FPNS) system was developed and demonstrated as a new tool for dynamically measuring nectar volume and concentration in artificial flowers. Experimental validation showed its ability to detect gradual variations in nectar volume and changes influenced by evaporation under controlled conditions. These results indicate how environmental factors, such as temperature and humidity, influence nectar availability, suggesting the value of integrating additional sensors for modeling and predicting these dynamics. This approach supports replicating natural scenarios more closely and adjusting nectar availability to optimize pollinator rewards. The FPNS’s capabilities present new avenues for investigating pollinator–flower interactions and designing more effective and controlled experiments. The FPNS is designed to be easily replicated to design flowerpatches with multiple flowers. In particular, such integrated sensing will provide a basis for future research on more precise neuroscience assays to investigate how dynamic nectar changes affect pollinator behavior and their relationship with the environment.

## Data Availability

The original contributions presented in this study are included in the article. Further inquiries can be directed to the corresponding authors.

## Abbreviations

The following abbreviations are used in this manuscript:

FPNS	Flower Patch Nectar Sensor
PCB	Printed Circuit Board
IC	Integrated Circuit
RH	relative humidity
VCC	Voltage Supply
GND	ground
μL	Microliter
mL	Milliliter
μF	Microfarad
nF	Nanofarad
kΩ	Kiloohm
Hz	Hertz
